# Characterization of PLA/LW-PLA Composite Materials Manufactured by Dual-Nozzle FDM 3D-Printing Processes

**DOI:** 10.3390/polym16202852

**Published:** 2024-10-10

**Authors:** Ye-Eun Park, Sunhee Lee

**Affiliations:** 1 Department of Fashion and Textiles, Dong-A University, Busan 49315, Republic of Korea; parkyeeun929@gmail.com; 2 Department of Fashion Design, Dong-A University, Busan 49315, Republic of Korea

**Keywords:** lightweight polylactic acid, composite materials, dual-nozzle extrusion, fused deposition modeling (FDM) 3D printing, compressive property

## Abstract

This study investigates the properties of 3D-printed composite structures made from polylactic acid (PLA) and lightweight-polylactic acid (LW-PLA) filaments using dual-nozzle fused-deposition modeling (FDM) 3D printing. Composite structures were modeled by creating three types of cubes: (i) ST4—built with a total of four alternating layers of the two filaments in the *z*-axis, (ii) ST8—eight alternating layers of the two filaments, and (iii) CH4—a checkered pattern with four alternating divisions along the *x*, *y*, and *z* axes. Each composite structure was analyzed for printing time and weight, morphology, and compressive properties under varying nozzle temperatures and infill densities. Results indicated that higher nozzle temperatures (230 °C and 240 °C) activate foaming, particularly in ST4 and ST8 at 100% infill density. These structures were 103.5% larger on one side than the modeled dimensions and up to 9.25% lighter. The 100% infill density of ST4-Com-PLA/LW-PLA-240 improved toughness by 246.5% due to better pore compression. The ST4 and ST8 cubes exhibited decreased stiffness with increasing temperatures, while CH4 maintained consistent compressive properties across different conditions. This study confirmed that the characteristics of LW-PLA become more pronounced as the material is printed continuously, with ST4 showing the strongest effect, followed by ST8 and CH4. It highlights the importance of adjusting nozzle temperature and infill density to control foaming, density, and mechanical properties. Overall optimal conditions are 230 °C and 50% infill density, which provide a balance of strength and toughness for applications.

## 1. Introduction

Additive manufacturing (AM), commonly referred to as 3D printing, is a process of creating three-dimensional objects by slicing objects layer-by-layer, as opposed to traditional subtractive manufacturing methods [1,2]. One of the most widely utilized AM technologies is fused-deposition modeling (FDM) 3D printing, which involves the extrusion of thermoplastic filaments to build objects [3,4]. FDM 3D printing for composite materials benefits from using dual extrusion nozzles, and it not only offers the advantages of dispositioning two different types of materials simultaneously, but it can also reduce printing time by up to 80% while maintaining surface quality. This dual-nozzle extrusion enables the creation of composite structures that combine the properties of different materials to achieve enhanced mechanical and functional properties. The range of available filaments for FDM 3D printing is becoming more diverse. Also, among the types of materials available, polymers and fiber-reinforced polymer composites are widely used due to their properties of high strength, low density, ease of manufacturing, and reduced manufacturing costs. It can enable the fabrication of composite materials with parts of superior strength and tailored properties [5,6,7,8,9]. The mechanical properties of these multi-material 3D-printed objects are heavily influenced by the quality of the interface formed between different materials. The nature of this interface, which is created at the junction where different materials meet, depends on factors such as the intrinsic properties of the materials used and the specific 3D-printing conditions under which the 3D-printing process is conducted [6,10].

Porous polymers, often classified as low-density materials, are characterized by the presence of numerous small, closed-cell pores distributed throughout the polymer matrix. These polymers form a foam structure as the chemical blowing agent within the matrix dissolves due to thermodynamic instability caused by a drop in pressure or an increase in temperature. These materials have attracted significant attention due to their structural properties, which lead to various enhanced mechanical, physical, and thermal characteristics [11,12,13,14,15,16]. One of the advanced methods for manufacturing porous polymers is FDM 3D-printing technology. This AM process enables precise control over the pore structure and distribution within the polymer, allowing for the customization of material properties to meet specific application requirements. Porous polymers exhibit excellent energy absorption properties, making them suitable for impact-resistant applications where high shock absorption is crucial. Additionally, they offer exceptional properties of sound absorption, fracture strength, fracture toughness, and thermal insulation [17,18,19,20].

In FDM 3D-printing technology, the quality of porous polymers can be controlled using slicing software. The most suitable material properties for a 3D object are achieved through the use of slicing software such as Cura, Prusa, or Cubicreator. These programs allow for the adjustment of various parameters that significantly impact the quality of the printed object. The parameters that can be adjusted within the slicing software include nozzle temperature, bed temperature, flow rate, layer height, infill pattern, and density, etc. The foam structure of porous polymers, which is influenced by thermodynamic instability, is an important characteristic that can control the flow rate and nozzle temperature through the slicing software [21,22,23,24,25]. Yousefi Kanani [16] and Damanpack [21] investigated the foaming characteristics of porous polylactic acid (PLA) using flow rate and nozzle temperature as parameters. Their study indicated that, as the nozzle temperature increased, the foaming characteristics became more pronounced, resulting in a decrease in density. Conversely, as the flow rate increased, the density also increased. Thus, this research investigated the foaming characteristics of porous PLA by fixing the flow rate and setting various nozzle temperatures with composite structures.

Previous studies have actively evaluated the mechanical properties of 3D-printed prototypes manufactured under various parameters by FDM 3D-printing technology [26,27,28,29,30]. In Chen [31], three-pointed star-shaped auxetic structures with different thicknesses and samples were manufactured using lightweight thermoplastic polyurethane (TPU) by FDM 3D-printing process conditions. They checked morphologies, specific gravities, thermal properties, and compression characteristics under the nozzle temperatures of 200 °C, 220 °C, and 240 °C. It was confirmed that as the nozzle temperature increased, the compressive deformation rates decreased, and the compressive strength increased. Based on that study [31], Chen [32] demonstrated the physical properties of multiple outsole designs of 3-, 4-, and 6-pointed star-shaped patterns and various thicknesses for 5, 7.5, and 10 mm. The physical and foot-pressure distribution properties were evaluated to confirm the best quality and comfort outsole. This study shares similarities with the previous study in that it involved a 3D-printing process using a porous polymer. However, the difference lies in the use of composite structure and in controlling the properties by applying additional materials to the composite structure.

The purpose of the present study is to develop composite structures using PLA and LW-PLA filaments through a dual-nozzle FDM 3D-printing process and to analyze their mechanical properties. To confirm this, three types of composite structures were used: ST4, which consists of four alternating layers of the two filaments along the *z*-axis; ST8, with eight alternating layers of the two filaments; and CH4, featuring a checkered pattern with four alternating divisions along the *x*, *y*, and *z* axes. The 3D-printing conditions were set at 200 °C for PLA and 220 °C to 240 °C for LW-PLA, with infill densities ranging from 25% to 100%.

First, the printing time, weight, and morphology of the 3D-printed composite structures were analyzed to confirm the variations resulting from each parameter and to compare the differences between the structures. Second, the compression characteristics of each structure were evaluated to investigate the mechanical properties of the filaments within single structures and to compare the characteristics between structures with different patterns. This study can enhance the mechanical properties by simultaneously addressing both material and manufacturing processes. It evaluated composite structures that use the same amount of functional material from various aspects. Its results are foundational data and are intended to be applied to 3D-printed arch pads for flat feet.

## 2. Experimental Section

### 2.1. Materials

Table 1 and Table 2 show the materials used. A dual-nozzle FDM 3D printer and two types of filaments, polylactic acid (PLA) and lightweight PLA (LW-PLA), were used for analysis. Firstly, the dual-nozzle FDM 3D printer used was the Ultimaker S5 Pro Bundle (Ultimaker B.V., Geldermalsen, The Netherlands), which is compatible with two 0.4 mm nozzles and 2.85 mm diameter filaments. Next, the PLA filament (Ultimaker B.V., Geldermalsen, The Netherlands) used had a diameter and hardness of 2.85 mm and 83 D and a density of 1.24 g/mm^3^. The LW-PLA filament (colorFabb B.V., Belfeld, The Netherlands) had the same diameter and density but a hardness of 95 A.

### 2.2. Sample Preparation

#### 2.2.1. D Modeling of PLA/LW-PLA Composite Structures

Table 3 depicts the 3D modeling of PLA/LW-PLA composite structures developed using PLA and LW-PLA filaments. The three types of cubes were developed using Fusion 360 v.2.0.20460 (Autodesk, Inc., San Francisco, CA, USA) 3D-modeling software, maintaining the same cube size. First, a striped pattern was divided into four along the *z*-axis, producing one object sized 10 × 10 × 2.5 mm^3^. Second, a striped type was divided into eight along the *z*-axis, resulting in one object sized 10 × 10 × 1.25 mm^3^. Lastly, a checkered type was divided into four along the *x*, *y*, and *z* axes, yielding one object sized 1.25 × 1.25 × 1.25 mm^3^. These three types of objects are categorized as follows: striped pattern with *z*-axis division by 4 (ST4), striped pattern with *z*-axis division by 8 (ST8), and checkered pattern with division along *x*, *y*, and *z* axes by 4 (CH4). These 3D objects were saved as *.stl files to enable slicing for cross-filament printing.

#### 2.2.2. D-Printing Conditions of PLA/LW-PLA Composite Structures

Table 4 and Table 5 contain the slicing and 3D-printing conditions for the PLA/LW-PLA composite structures. Using the Ultimaker Cura 5.2.1 (Ultimaker B.V., Geldermalsen, The Netherlands) of slicing software, each nozzle of the dual nozzle was individually controlled. First, filaments were assigned to each nozzle: Nozzle 1 was designated for PLA filament, and Nozzle 2 for LW-PLA filament. Each cube was sliced into 50 layers, and the 3D-printing conditions for the dual nozzle were set as follows: for Nozzle 1 handling PLA, the nozzle temperature was set to 200 °C, the bed temperature to 60 °C, and the printing speed to 60 mm/s, with zigzag infill patterns at 25%, 50%, 75%, and 100% infill density. For Nozzle 2 handling LW-PLA, the nozzle temperature was incrementally set from 220 °C to 240 °C in 10 °C intervals, maintaining consistent conditions for analysis. Each sample was designated with a sample code (structure type–Com–PLA/LW-PLA–nozzle temperature–infill density) and converted into a printable g-code file. 

### 2.3. Characterization

#### 2.3.1. Actual Printing Time and Weight

The printing time and weight of each 3D-printed PLA/LW-PLA composite structure were verified. The printing time was measured from the time displayed on the 3D printer after completion of printing. Units used were minutes (m) and seconds (s). The weight was measured using electronic balances (PAG114, Ohaus, Parsippany, NJ, USA). The unit used was grams (g). Each sample was printed and measured at least three times, and the average values of three reliable specimens were used for result analysis. Based on the size of the sample and weight, the density was confirmed. The mass-per-unit volume (g/mm^3^) of a substance is called density. Density is calculated by dividing the weight by the volume.

#### 2.3.2. Morphology

To analyze the surface characteristics of the 3D-printed PLA/LW-PLA composite structures, the fabric image analysis system (NTZ-6000, Nextecvision Co., Ltd., Anyang-si, Gyeonggi-do, Republic of Korea) was utilized. The fabric image analysis system was employed at a magnification of × 4.55 for surface analysis of the three types of PLA/LW-PLA composite structures.

#### 2.3.3. Compressive Property

**Figure 1:**
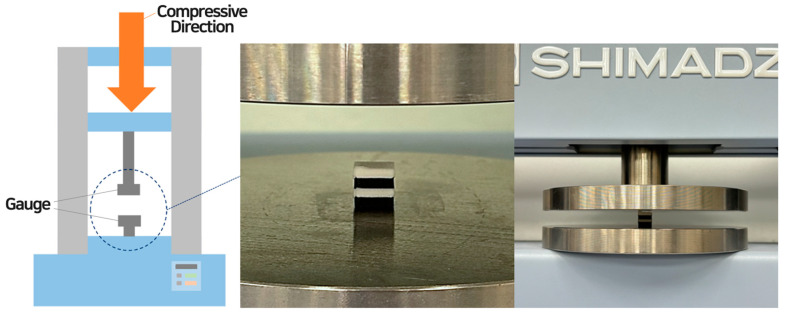
Universal mechanical testing machine for compressive property of 3D-printed cubes using PLA and LW-PLA filaments.

To verify the compressive properties of the 3D-printed samples, the measurements in Figure 1 were conducted using a universal testing machine (AGS-X, Shimadzu, Nakagyo-ku, Kyoto, Japan) with a 5 kN load cell, following the KS M ISO 604 Plastics—Determination of Compressive Properties [33]. The sample dimensions were 10 × 10 × 10 mm^3^, with a gauge height of 10 mm, and each sample was centrally positioned in the testing machine. The compression rate was set to 10 mm/min, and each test was repeated three times to obtain average values for analysis. Parameters measured included the compressive stress–strain curve, initial compressive modulus, maximum compressive stress, maximum compressive strain, and compressive toughness.

## 3. Results and Discussion

### 3.1. Actual Printing Time and Weight of 3D-Printed PLA/LW-PLA Composite Structures

**Figure 2:**
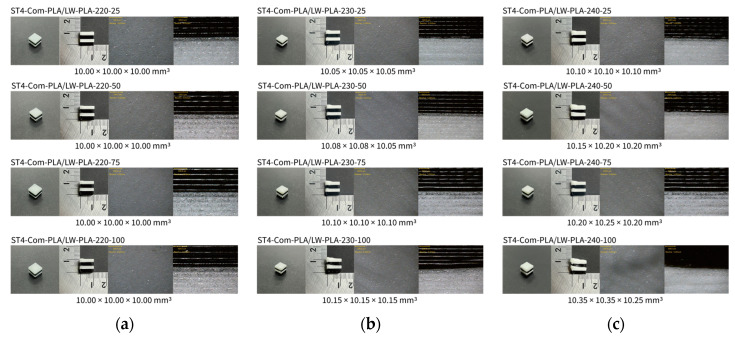
Three dimensional-printed ST4-Com-PLA/LW-PLA with various nozzle temperatures and infill densities: (**a**) 220 °C, (**b**) 230 °C, and (**c**) 240 °C.

**Figure 3:**
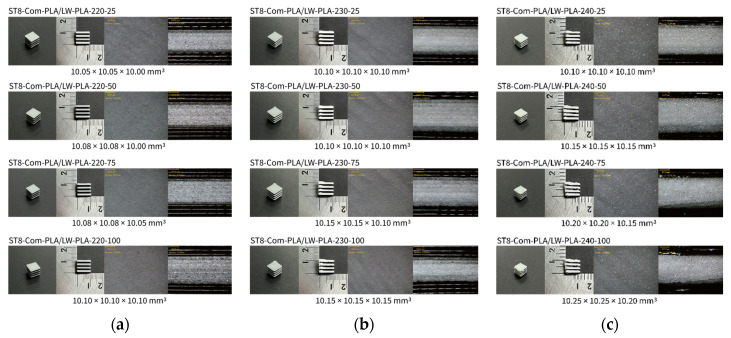
Three dimensional-printed ST8-Com-PLA/LW-PLA with various nozzle temperatures and infill densities: (**a**) 220 °C, (**b**) 230 °C, and (**c**) 240 °C.

**Figure 4:**
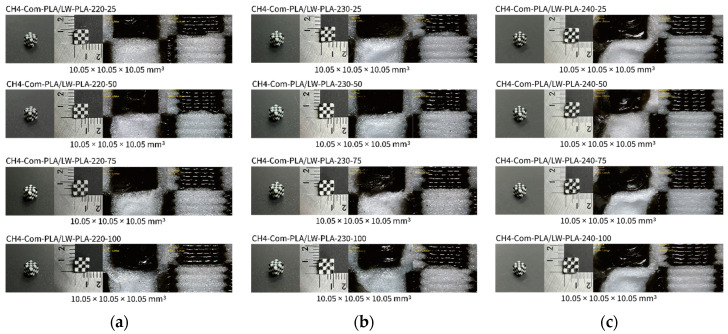
Three dimensional-printed CH4-Com-PLA/LW-PLA with various nozzle temperatures and infill densities: (**a**) 220 °C, (**b**) 230 °C, and (**c**) 240 °C.

**Figure 5:**
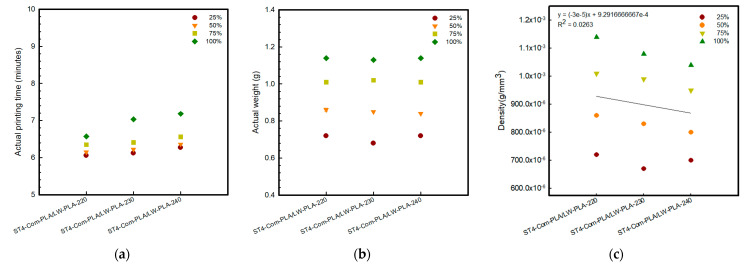
Actual printing time and weight of 3D−printed ST4−Com−PLA/LW−PLA with various nozzle temperatures and infill densities: (**a**) actual printing time, (**b**) actual weight, and (**c**) density.

**Figure 6:**
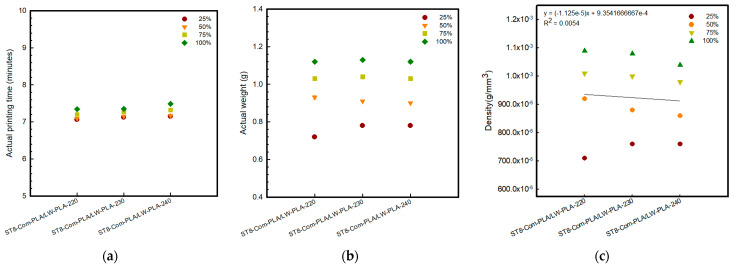
Actual printing time and weight of 3D−printed ST8−Com−PLA/LW−PLA with various nozzle temperatures and infill densities: (**a**) actual printing time, (**b**) actual weight, and (**c**) density.

**Figure 7:**
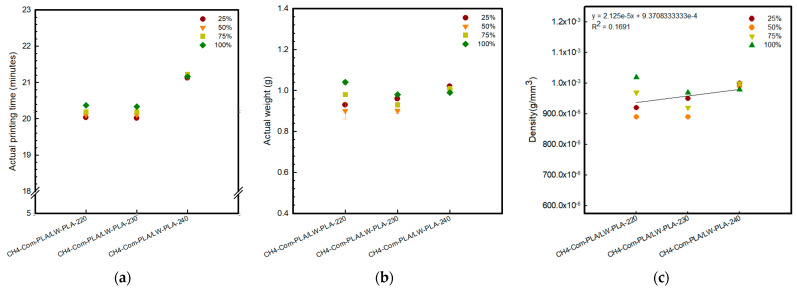
Actual printing time and weight of 3D−printed CH4−Com−PLA/LW−PLA with various nozzle temperatures and infill densities: (**a**) actual printing time, (**b**) actual weight, and (**c**) density.

Figure 2, Figure 3 and Figure 4 present 3D-printed composite structures: ST4-Com-PLA/LW-PLA, ST8-Com-PLA/LW-PLA, and CH4-Com-PLA/LW-PLA. Figure 5, Figure 6 and Figure 7 show the actual printing time, weight, and density. 

When checking the printing time for the ST4-Com-PLA/LW-PLA-220 sample, the times were confirmed as 6 min 06 s, 6 min 15 s, 6 min 35 s, and 6 min 57 s for infill densities of 25%, 50%, 75%, and 100%, respectively. Compared to 220 °C, each 10 °C increase required an additional 6 s and 21 s, respectively. For ST8-Com-PLA/LW-PLA, the printing time varied with nozzle temperature increases, but no clear pattern of increase or decrease was observed. Specifically, the printing times for ST8-Com-PLA/LW-PLA-220 were 7 min 06 s at 25% infill density, 7 min 09 s at 50%, 7 min 20 s at 75%, and 7 min 34 s at 100%. Increasing the nozzle temperature by 10 °C for ST8-Com-PLA/LW-PLA-220 resulted in approximately 6 s longer at 230 °C and between 8 to 15 s longer at 240 °C. The printing time for CH4-Com-PLA/LW-PLA significantly increased compared to other composites, confirmed to be around 20–21 min. For CH4-Com-PLA/LW-PLA-220, the printing times were 20 min 03 s, 20 min 10 s, 20 min 19 s, and 20 min 37 s as the infill density increased by 25%. Samples printed at 240 °C took approximately 1 min longer than those printed at 220 °C, with about a 1 min difference. However, there was no significant difference observed at 230 °C.

The composite was modeled as a hexagon with a side length of 10 mm. For ST4-Com-PLA/LW-PLA-220, the printed size matched the modeled dimensions regardless of infill density. As the infill density increased, its weight increased from 0.72 g at 25% infill density to 1.14 g at 100%, a 1.58-times increase. For ST4-Com-PLA/LW-PLA-230, the side length increased by 0.05 mm at 25% infill density and by 0.15 mm at 100%. The weight was 0.68 g at 25% infill density and 1.13 g at 100%, a 1.66-times increase. Despite the increased volume, the weight was similar to ST4-Com-PLA/LW-PLA-220, indicating a lower density for the composite structure. ST4-Com-PLA/LW-PLA-240 showed an even greater volume increase, with the side length increasing by 0.10 mm at 25% infill density and by 0.35 mm at 100%. The weight checked the same as ST4-Com-PLA/LW-PLA-220, confirming it as the least-dense composite structure. As the nozzle temperature increased, the volume of the composite structure also increased. This can be attributed to the foaming characteristics of the filament, where bubbles form during extrusion, increasing volume while maintaining low weight. It was confirmed that the LW-PLA filament exhibited prominent foaming characteristics as the nozzle temperature increased; foaming characteristics did not appear at 200 °C, and foaming characteristics began to appear at 220 °C [34].

For ST8-Com-PLA/LW-PLA, the volume increased compared to ST4-Com-PLA/LW-PLA at nozzle temperatures of 220 °C and 230°. For ST8-Com-PLA/LW-PLA-220, 230, and 240, the side length increased by 0.05 mm, 0.10 mm, and 0.10 mm at 25% infill density and by 0.10 mm, 0.15 mm, and 0.25 mm at 100% infill density, respectively. The weight of ST8-Com-PLA/LW-PLA at different nozzle temperatures was confirmed to be 0.72–0.78 g at 25% infill density, 0.90–0.93 g at 50%, 1.03–1.04 g at 75%, and 1.12–1.13 g at 100%. The weight increased 1.45–1.56 times from 25% to 100% infill density. ST8-Com-PLA/LW-PLA also exhibited increased volume than the modeling dimension. When compared to ST4-Com-PLA/LW-PLA, the weights of ST8-Com-PLA/LW-PLA-220 and 230 were similar, but the volume of ST4-Com-PLA/LW-PLA was smaller, resulting in a higher density for ST8-Com-PLA/LW-PLA. However, ST8-Com-PLA/LW-PLA-240 had a smaller volume than ST4-Com-PLA/LW-PLA-240, indicating a lower density for ST8-Com-PLA/LW-PLA-240.

In the case of CH4-Com-PLA/LW-PLA, all composite structures showed the same volume, which increased by 0.05 mm compared to the intended modeling. The weight ranged from 0.93 to 1.04 g, with no significant variation between nozzle temperatures or infill densities. However, the weight of CH4-Com-PLA/LW-PLA-240 ranged from 0.99 to 1.02 g regardless of infill densities, which was higher than at other nozzle temperatures. In this composite structure, the LW-PLA filament was not printed continuously but intersected with another filament within one layer. Accordingly, it is considered that the overall higher density is due to the reduced foaming area.

Therefore, as the nozzle temperature increases, the foaming characteristics of the LW-PLA filament become more pronounced, especially when printed continuously. This suggests that by adjusting the nozzle temperature and infill density during the 3D-printing process, it is possible to effectively control the density and volume of the composite structure.

### 3.2. Morphology of 3D-Printed PLA/LW-PLA Composite Structures

Figure 2 illustrates the morphology of ST4-Com-PLA/LW-PLA, highlighting the interfacial boundaries between the PLA and LW-PLA filaments. Figure 3 shows the morphology of ST8-Com-PLA/LW-PLA, while Figure 4 presents the morphology of CH4-Com-PLA/LW-PLA. FDM 3D printers print samples layer-by-layer. In the ST4-Com-PLA/LW-PLA and ST8-Com-PLA/LW-PLA, one nozzle is used per layer, and two nozzles are used alternately between layers. In contrast, the CH4-Com-PLA/LW-PLA uses two nozzles per layer, and the two nozzles are used alternately within each layer. Regardless of the type of composite structures, dual-nozzle 3D printing with PLA and LW-PLA filaments demonstrates a stable printing state without problems such as interlayer delamination or nozzle clogging at the interface between the materials.

As previously mentioned, it was confirmed that ST4-Com-PLA/LW-PLA showed increased foaming and dimensional expansion with increasing nozzle temperature and infill density. However, ST4-Com-PLA/LW-PLA-220 exhibited no dimensional change across different infill densities. ST4-Com-PLA/LW-PLA-220 had relatively smooth surfaces with minimal foaming. In contrast, ST4-Com-PLA/LW-PLA-230 showed an increase in the amount of foaming and surface roughness with higher infill densities. ST4-Com-PLA/LW-PLA-240 exhibited a similar trend, with even more foaming and rougher surfaces as the infill density increased. Unlike ST4-Com-PLA/LW-PLA, ST8-Com-PLA/LW-PLA showed dimensional expansion starting from ST8-Com-PLA/LW-PLA-220, but the rate of increase was less for ST8-Com-PLA/LW-PLA-240. This composite structure exhibits similar surface morphology trends with variations in ST4-Com-PLA/LW-PLA.

When examining a single structure within the ST4 and ST8-Com-PLA/LW-PLA, it is evident that foaming becomes more active as the layer height increases for ST4 and ST8-Com-PLA/LW-PLA-230 and 240 printed with LW-PLA. This is due to the nozzle scraping each layer as it is deposited, enhancing the foaming process. It can be confirmed in the top view of the morphology, and the scratch phenomenon of the nozzle can be confirmed. As the infill density increases, foaming improves because the filament is extruded more densely due to the foaming characteristics compared to non-foaming filaments. Also, the extruded filaments overlap, causing more increase in overall dimensions. Therefore, PLA filaments inevitably overlap and print together with structures printed with LW-PLA. ST8-Com-PLA/LW-PLA exhibits fewer foaming properties compared to ST4-Com-PLA/LW-PLA.

In the case of CH4-Com-PLA/LW-PLA, dual nozzles were applied along the x, y, and z axes, enabling printing both across layers and within a single layer. This method results in lower foaming activity compared to ST4-Com-PLA/LW-PLA and ST8-Com-PLA/LW-PLA due to the triaxle division and smaller overall cube size of 1.25 × 1.25 × 1.25 mm^3^. Consequently, the differences in infill densities are less pronounced in the morphological images. However, as the object size increases, the differences according to infill parameters are expected to become more evident. Despite the smaller size, an examination of the printed structures revealed that as the nozzle temperature increases, the overlap between different filaments expands more significantly. This indicates that the foaming and interaction between PLA and LW-PLA filaments become more pronounced at higher nozzle temperatures, even in smaller CH4-Com-PLA/LW-PLA.

Based on this, the density was evaluated in terms of weight per volume of each composite structure. In the case of ST4-Com-PLA/LW-PLA, the volume expanded while the weight remained similar across different temperatures, confirming an overall decrease in density by up to 9.25%. For ST8-PLA/LW-PLA, although the density also decreased overall, the reduction was up to 4.5%. This indicated the lightweight property of LW-PLA, but the decrease was significantly lower than ST4-Com-PLA/LW-PLA. In the case of CH4, densities were generally similar and consistent, with CH4-Com-PLA/LW-PLA-240 showing the closest infill density. The density exhibited a pattern of increasing as the temperature increased. Therefore, it was confirmed that LW-PLA demonstrated foaming properties as the range of continuous printing increased.

### 3.3. Compressive Property of 3D-Printed PLA/LW-PLA Composite Structures

**Figure 8:**
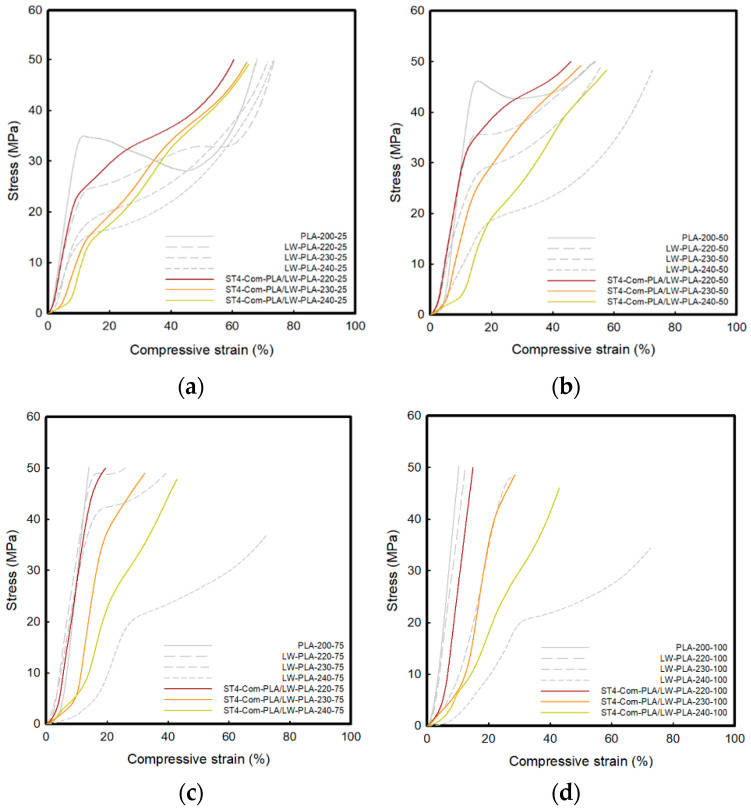
Compressive S–S curve of ST4-Com-PLA/LW-PLA with various temperatures and infill densities: (**a**) 25% infill density, (**b**) 50% infill density, (**c**) 75% infill density, and (**d**) 100% infill density.

**Figure 9:**
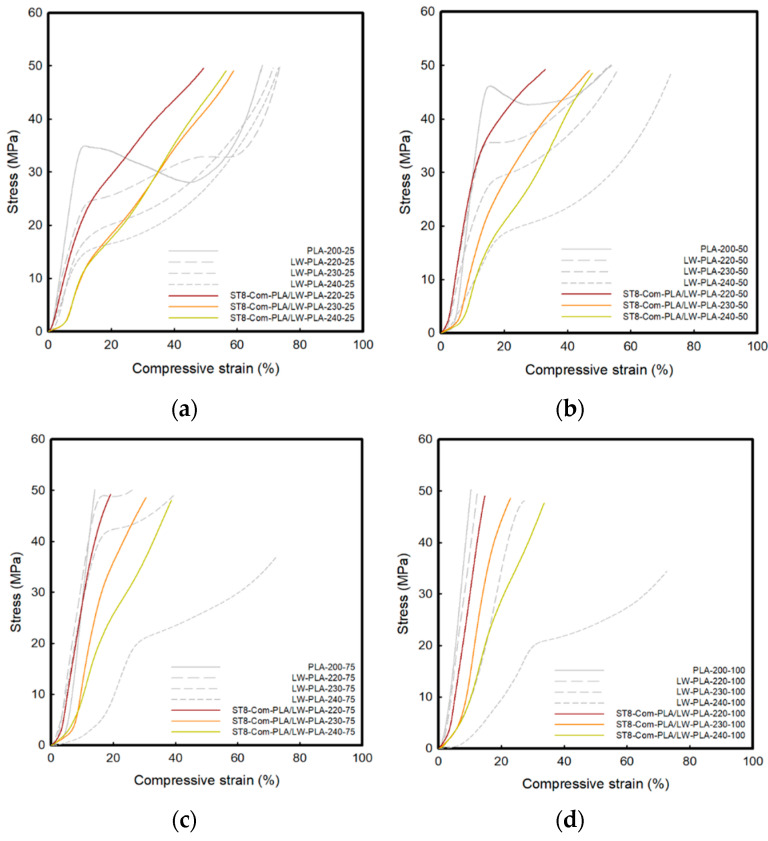
Compressive S–S curve of ST8-Com-PLA/LW-PLA with various temperatures and infill densities: (**a**) 25% infill density, (**b**) 50% infill density, (**c**) 75% infill density, and (**d**) 100% infill density.

**Figure 10:**
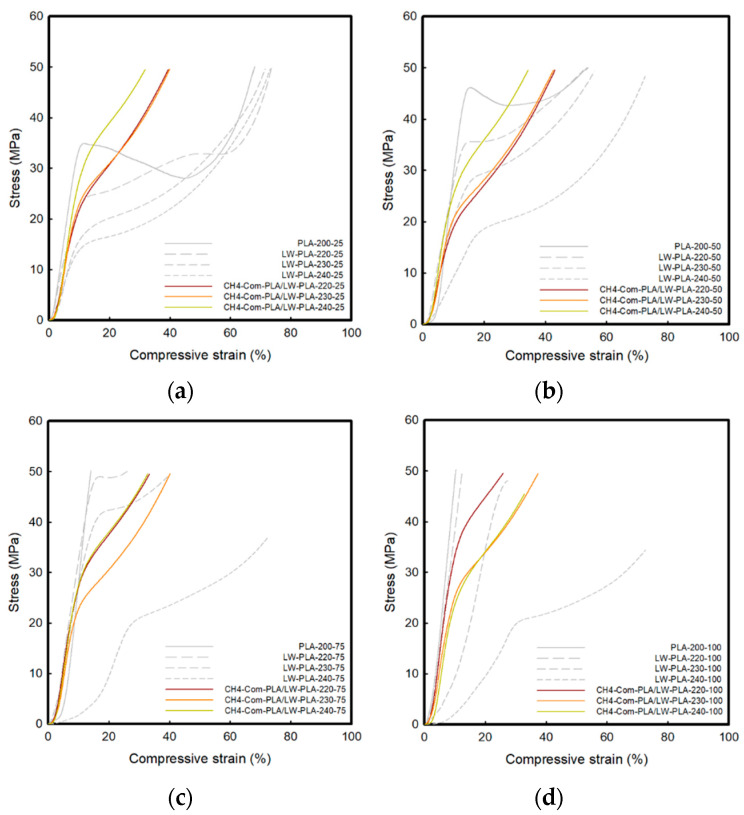
Compressive S–S curve of CH4-Com-PLA/LW-PLA with various temperatures and infill densities: (**a**) 25% infill density, (**b**) 50% infill density, (**c**) 75% infill density, and (**d**) 100% infill density.

Figure 8, Figure 9 and Figure 10 show the compressive stress–strain (S–S) curves for three types of Com-PLA/LW-PLA at various nozzle temperatures and infill densities. Also, Table 6, Table 7 and Table 8 present the compressive properties. The compressive S–S curves for the three types of Com-PLA/LW-PLA exhibit a tendency for stress to continuously increase with increasing strain. The initial modulus of ST4-Com-PLA/LW-PLA-220 ranged from 234.37 MPa at 25% infill density to 263.17 MPa at 100% infill density. For ST4-Com-PLA/LW-PLA-230, the initial modulus ranged from 112.42 MPa at 25% infill density to 68.05 MPa at 100% infill density. Additionally, the initial modulus of ST4-Com-PLA/LW-PLA-240 spanned from 79.18 MPa at 25% infill density to 63.31 MPa at 100% infill density. The initial modulus of ST4-Com-PLA/LW-PLA decreased as the nozzle temperature increased from 220 °C to 240 °C. The highest initial modulus was observed at 220 °C, followed by 230 °C, with the lowest at 240 °C. This shows that the stiffness of the filament decreases as the foaming phenomenon becomes more active at high nozzle temperatures.

At 50% compression, the compressive strength of the samples, except for those with 25% infill density, reached a maximum value. However, ST4-Com-PLA/LW-PLA-240-50 showed a lower value of 43.91 MPa at the same infill density. This suggests that the composite structures become less stiff and exhibit more flexible compression behavior as the nozzle temperature rises. The LW-PLA filament becomes more flexible, and the compressive strength decreases as the nozzle temperature increases, due to more active foaming at high nozzle temperatures and reduced compressive strength.

The toughness of ST4-Com-PLA/LW-PLA tended to decrease as the nozzle temperature increased, but it increased with higher infill density. For 25% infill density, the toughness ranged from 19.14 J to 16.99 J, and for 50% infill density, it ranged from 16.15 J to 14.64 J, both decreasing with nozzle temperature. However, for 75% and 100% infill density, the toughness increased with higher nozzle temperatures, with values of 4.96 J and 2.71 J at 220 °C, 8.12 J and 6.03 J at 230 °C, and 9.99 J and 9.39 J at 240 °C, respectively. This increase in toughness at higher infill densities is due to the greater compression of bubbles, which enhances their energy absorption capacity.

As the temperature increased, the initial modulus of ST8-Com-PLA/LW-PLA at 25% infill density was 198.05 MPa at 220 °C, 94.36 MPa at 230 °C, and 96.94 MPa at 240 °C. At 100% infill density, the initial modulus was 297.93 MPa at 220 °C, 138.5 MPa at 230 °C, and 93.31 MPa at 240 °C. Similar to ST4-Com-PLA/LW-PLA, the initial elastic modulus decreased with increasing temperature, but the values at the lower temperature (220 °C) were consistently higher than those at higher temperatures (230 °C and 240 °C).

The compressive strength at 50% for ST8-Com-PLA/LW-PLA with 25% infill density was 41.67 MPa at 230 °C and 43.66 MPa at 240 °C. Apart from these values, ST8-Com-PLA/LW-PLA exhibited maximum strength. In general, ST8-Com-PLA/LW-PLA demonstrated higher compressive strength compared to ST4-Com-PLA/LW-PLA. This indicates that the ST4-Com-PLA/LW-PLA has structural properties that allow it to maintain higher strength.

For 25% and 50% infill density, the toughness was 15.34 J and 10.95 J for ST8-Com-PLA/LW-PLA-220, 15.04 J and 13.47 J for ST8-Com-PLA/LW-PLA-230, and 14.09 J and 11.68 J for ST8-Com-PLA/LW-PLA-240, respectively. The toughness generally decreased as the temperature increased. At higher infill densities (75% and 100%), the toughness ranged from 4.67 J and 3.05 J for ST8-Com-PLA/LW-PLA-220 to 9.28 J and 7.83 J for ST8-Com-PLA/LW-PLA-240. At these higher infill densities, ST8-Com-PLA/LW-PLA-240 showed higher toughness compared to ST8-Com-PLA/LW-PLA-220. Overall, ST8-Com-PLA/LW-PLA exhibited different mechanical properties, particularly in terms of strength and toughness, compared to ST4-Com-PLA/LW-PLA. ST8-Com-PLA/LW-PLA generally showed a lower initial modulus, higher compressive strength, and greater toughness at higher temperatures and infill densities.

Unlike other composite structures, CH4-Com-PLA/LW-PLA showed relatively little variation among parameters such as temperatures and infill densities. The initial modulus ranged from 187.75 MPa to 275.30 MPa. This indicates that CH4-Com-PLA/LW-PLA has high resistance to temperature changes and little change in mechanical strength due to foaming. CH4-Com-PLA/LW-PLA exhibited maximum strength at all infill densities. The toughness of the samples was 8.30–12.00 J. In toughness, it maintained consistent strength and was similar to the 50% infill density of ST8-Com-PLA/LW-PLA.

The compressive properties of LW-PLA filaments with the three types of composite structures are highly dependent on temperature and infill density, and the foaming phenomenon plays an important role. ST4 and ST8- Com-PLA/LW-PLA show a decrease in stiffness and toughness due to foaming at high temperatures, but the compressive properties tend to improve with increasing infill density. On the other hand, CH4-Com-PLA/LW-PLA shows less variation in compressive properties with temperature and infill density changes, providing more consistent performance. 

For the three composite structures, at the intermediate extrusion temperature of 230 °C, foaming occurs appropriately, leading to balanced compression properties. Additionally, both strength and toughness are relatively well-balanced, and the formation and compression of bubbles are considered optimal. Regarding infill density, 50% infill density provides an adequate balance between strength and toughness. However, if toughness is particularly important, a higher infill density should be considered. Therefore, this study recommends the use of Com-PLA/LW-PLA-230-50 for the three composite structures. However, depending on the application, the temperature and density can be adjusted for optimal performance.

## 4. Conclusions

This study confirms the properties of 3D-printed composite structures made from PLA and LW-PLA filaments by dual-nozzle FDM 3D printing. The research focused on three different composite structures with various nozzle temperatures and infill densities: ST4-Com-PLA/LW-PLA, ST8-Com-PLA/LW-PLA, and CH4-Com-PLA/LW-PLA. It analyzed their printing time and weight, morphologies, and compressive properties.

- For ST4-Com-PLA/LW-PLA, printing time increased with both nozzle temperature and infill density. Notably, higher nozzle temperatures (230 °C and 240 °C) activated foaming; these structures were 103.5% larger on one side than the modeled dimensions and up to 9.25% lighter. The 100% infill density of ST4-Com-PLA/LW-PLA-240 improved toughness by 246.5% due to better pore compression. ST8-Com-PLA/LW-PLA showed a similar pattern in printing time variations but without a clear trend related to nozzle temperature. The volume also increased with temperature, leading to variable densities. CH4-Com-PLA/LW-PLA exhibited the longest printing times and the highest weights compared to other structures. The structure showed minimal weight variation across different parameters, suggesting a stable printing process.
- Foaming characteristics became more pronounced at higher temperatures, especially in ST4-Com-PLA/LW-PLA and ST8-Com-PLA/LW-PLA. ST4-Com-PLA/LW-PLA displayed significant foaming and dimensional expansion at higher temperatures, resulting in rougher surfaces. ST8-Com-PLA/LW-PLA showed less dimensional expansion compared to ST4 but exhibited similar trends in surface morphology. CH4-Com-PLA/LW-PLA had the least foaming activity and minimal dimensional change, indicating stable morphology across different conditions.
- ST4-Com-PLA/LW-PLA demonstrated a decrease in stiffness and toughness with increasing temperature, attributed to enhanced foaming. Higher infill densities improved toughness due to better bubble compression. ST8-Com-PLA/LW-PLA showed similar trends to ST4, with decreased stiffness at higher temperatures but generally higher compressive strength and toughness. CH4-Com-PLA/LW-PLA showed consistent compressive properties with little variation across different temperatures and infill densities, highlighting its resistance to temperature-induced changes.

The study highlights the significance of adjusting nozzle temperature and infill density to control the foaming characteristics, density, and compressive properties of PLA/LW-PLA composite structures. Specifically, ST4-Com-PLA/LW-PLA and ST8-Com-PLA/LW-PLA exhibit more significant changes in mechanical properties due to temperature variations. For applications, the composite structures printed at 230 °C with 50% infill density offer a balance between strength and toughness, making them suitable for a variety of uses. This study demonstrates the possibility of producing composite material 3D-printing prototypes using LW-PLA filament. The three types of structures developed can be applied to arch pads for flat feet, allowing for customization of properties according to the user. Additionally, a 3D-printed flat-feet product can be created with LW-PLA material on the inside to enhance energy absorption and a skin-friendly feel by printing TPU (thermoplastic polyurethane) material on the outside.

## Figures and Tables

**Table 1:** Filament specification.

Specification	Filament	
	PLA	LW-PLA
Company	Ultimaker B.V. (Geldermalsen, The Netherlands)	colorFabb B.V. (Belfeld, The Netherlands)
Color	Black	White
Diameter (mm)	2.85	2.85
Hardness	83 D	95 A
Density (g/mm^3^)	1.24	1.24
Recommended nozzle temperature (°C)	200	195–260
Recommended bed temperature (°C)	60	50–60
Recommended print speed (mm/s)	70	40–100

**Table 2:** 3D printer specification.

Specification	Dual-Nozzle FDM 3D Printer
	Ultimaker S5 Pro Bundle
Company	Ultimaker B.V. (Geldermalsen, The Netherlands)
Nozzle (mm)	AA 0.4 Dual-nozzle
Slicing software	Ultimaker Cura 5.2.1

**Table 3:** 3D modeling of PLA/LW-PLA composite structures.

	3D Modeling of Cube
Software	Fusion 360 v.2.0.20460 (Autodesk, Inc., San Francisco, CA, USA)
Size (cm^3^)	10 × 10 × 10
ST4	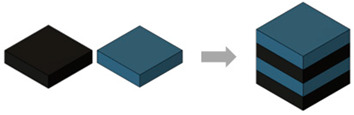
ST8	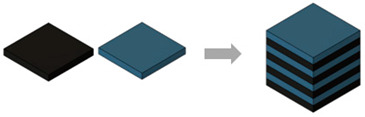
CH4	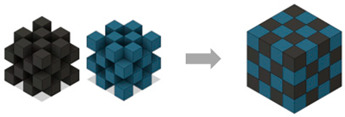

**Table 4:** Slicing of PLA/LW-PLA composite structures.

	Nozzle1	Nozzle2	Dual-Nozzle
	PLA	LW-PLA	PLA/LW-PLA
ST4	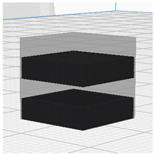	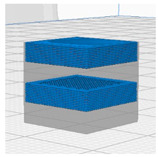	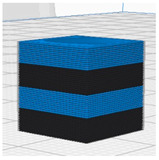
ST8	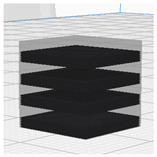	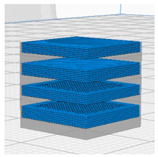	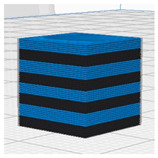
CH4	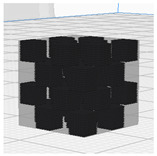	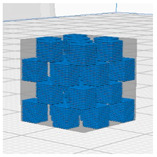	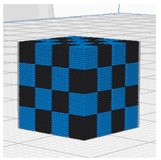

**Table 5:** 3D-printing conditions of PLA/LW-PLA composite structures.

	Nozzle1	Nozzle2
	PLA	LW-PLA
Nozzle (mm)	AA 0.4	
Nozzle temp. (°C)	200	220, 230, 240
Bed temp. (°C)	60	
Printing speed (mm/s)	70	
Infill pattern	Zigzag	
Infill density (%)	25, 50, 75, 100	

**Table 6:** Compressive properties of ST4-Com-PLA/LW-PLA with various temperatures and infill densities.

		Infill Density (%)			
		25	50	75	100
Initial modulus (MPa)	ST4-Com-PLA/LW-PLA-220	234.37 ± 2.83	281.06 ± 18.78	271.90 ± 12.72	263.17 ± 34.72
	ST4-Com-PLA/LW-PLA-230	112.42 ± 4.25	148.94 ± 12.31	56.55 ± 5.70	68.05 ± 2.05
	ST4-Com-PLA/LW-PLA-240	79.18 ± 3.30	36.56 ± 1.98	56.00 ± 1.45	63.31 ± 2.46
Stress at 50% (MPa)	ST4-Com-PLA/LW-PLA-220	41.04 ± 0.21	50.00 ± 0.00	50.00 ± 0.00	50.00 ± 0.00
	ST4-Com-PLA/LW-PLA-230	39.05 ± 0.17	50.00 ± 0.00	50.00 ± 0.00	50.00 ± 0.00
	ST4-Com-PLA/LW-PLA-240	38.22 ± 0.45	43.91 ± 0.27	50.00 ± 0.00	50.00 ± 0.00
Toughness (J)	ST4-Com-PLA/LW-PLA-220	19.14 ± 0.14	16.15 ± 0.48	4.96 ± 0.41	2.71 ± 0.05
	ST4-Com-PLA/LW-PLA-230	17.57 ± 0.29	14.84 ± 0.22	8.12 ± 0.37	6.03 ± 0.16
	ST4-Com-PLA/LW-PLA-240	16.99 ± 0.46	14.64 ± 0.48	9.99 ± 0.29	9.39 ± 0.10

**Table 7:** Compressive properties of ST8-Com-PLA/LW-PLA with various temperatures and infill densities.

		Infill Density (%)			
		25	50	75	100
Initial modulus (MPa)	ST8-Com-PLA/LW-PLA-220	198.05 ± 9.36	286.43 ± 13.96	274.95 ± 39.68	297.93 ± 23.00
	ST8-Com-PLA/LW-PLA-230	94.36 ± 4.06	129.47 ± 16.20	116.72 ± 6.49	138.54 ± 31.80
	ST8-Com-PLA/LW-PLA-240	96.94 ± 4.53	81.09 ± 2.05	90.27 ± 3.49	93.81 ± 3.66
Stress at 50% (MPa)	ST8-Com-PLA/LW-PLA-220	50.00 ± 0.00	50.00 ± 0.00	50.00 ± 0.00	50.00 ± 0.00
	ST8-Com-PLA/LW-PLA-230	41.67 ± 0.26	50.00 ± 0.00	50.00 ± 0.00	50.00 ± 0.00
	ST8-Com-PLA/LW-PLA-240	43.66 ± 0.66	50.00 ± 0.00	50.00 ± 0.00	50.00 ± 0.00
Toughness (J)	ST8-Com-PLA/LW-PLA-220	15.34 ± 0.12	10.95 ± 0.05	4.67 ± 0.61	3.05 ± 0.07
	ST8-Com-PLA/LW-PLA-230	15.04 ± 0.15	13.47 ± 0.02	7.56 ± 0.11	5.06 ± 0.10
	ST8-Com-PLA/LW-PLA-240	14.09 ± 0.03	11.68 ± 0.68	9.28 ± 0.07	7.83 ± 0.55

**Table 8:** Compressive properties of CH4-Com-PLA/LW-PLA with various temperatures and infill densities.

		Infill Density (%)			
		25	50	75	100
Initial modulus (MPa)	CH4-Com-PLA/LW-PLA-220	217.78 ± 1.99	187.75 ± 7.90	270.97 ± 8.78	332.43 ± 11.36
	CH4-Com-PLA/LW-PLA-230	229.02 ± 4.41	204.60 ± 2.16	227.50 ± 1.38	251.98 ± 6.72
	CH4-Com-PLA/LW-PLA-240	275.30 ± 5.27	251.19 ± 6.89	273.86 ± 17.85	235.98 ± 6.10
Stress at 50% (MPa)	CH4-Com-PLA/LW-PLA-220	50.00 ± 0.00	50.00 ± 0.00	50.00 ± 0.00	50.00 ± 0.00
	CH4-Com-PLA/LW-PLA-230	50.00 ± 0.00	50.00 ± 0.00	50.00 ± 0.00	50.00 ± 0.00
	CH4-Com-PLA/LW-PLA-240	50.00 ± 0.00	50.00 ± 0.00	50.00 ± 0.00	50.00 ± 0.00
Toughness (J)	CH4-Com-PLA/LW-PLA-220	11.45 ± 0.16	11.88 ± 0.08	10.38 ± 0.37	8.30 ± 0.34
	CH4-Com-PLA/LW-PLA-230	11.36 ± 0.21	12.00 ± 0.04	11.68 ± 0.07	11.38 ± 0.17
	CH4-Com-PLA/LW-PLA-240	9.87 ± 0.03	10.50 ± 0.10	10.15 ± 0.46	10.77 ± 0.09

## Data Availability

The original contributions presented in the study are included in the article, further inquiries can be directed to the corresponding author.
